# Predictors of Anxiety Trajectories in Cohort of First-Year College Students

**DOI:** 10.1016/j.jaacop.2024.08.004

**Published:** 2024-10-18

**Authors:** Laura S.P. Bloomfield, Mikaela Irene Fudolig, Julia N. Kim, Jordan Llorin, Juniper Lovato, Ellen W. McGinnis, Ryan S. McGinnis, Matthew Price, Taylor H. Ricketts, Peter Sheridan Dodds, Kathryn Stanton, Christopher M. Danforth

**Affiliations:** a University of Vermont, Burlington, Vermont; b Wake Forest University School of Medicine, Winston-Salem, North Carolina

**Keywords:** college students, mental health, generalized anxiety disorder, sleep, wearables

## Abstract

**Objective:**

The transition to college is a period of growth and vulnerability for young adult health and well-being and provides a critical window for potential behavioral interventions. In this study, we sought to examine the trajectory of anxiety symptoms and their association with individual characteristics, exposure to stressors, and sleep behaviors during the transition to college.

**Method:**

We recruited full-time, incoming undergraduate students at a university in the northeastern United States to participate during the first semester of college between October 21, 2022, and December 12, 2022. In a longitudinal cohort study (N = 556), we collected baseline demographic and health history information and weekly survey assessments with the outcome measure of anxiety. Predictors included weekly stressors and sleep measures during this period. Mixed-effects linear models were used to examine trajectories in anxiety symptoms during the first semester of college.

**Results:**

We had 6 main findings. First, there were significantly higher anxiety symptoms in non-male participants compared to male participants. Second, a previous mental health diagnosis and previous traumatic exposures were significant predictors of anxiety symptoms. Third, the personality traits of extraversion and neuroticism were significant predictors of anxiety symptoms. Fourth, perceived sleep duration, quality, and satisfaction were significant predictors of anxiety symptoms. Fifth, sleep duration estimates collected by a biometric wearable were also a significant predictor of anxiety in covariate-adjusted, corrected models. Sixth, weekly stressors and specifically academic stressors were significant predictors of anxiety symptoms.

**Conclusion:**

Programs that support young adults entering college may promote sleep hygiene behaviors and target times of particularly elevated stress such as examination periods.

**Diversity & Inclusion Statement:**

We worked to ensure that the study questionnaires were prepared in an inclusive way. One or more of the authors of this paper self-identifies as a member of one or more historically underrepresented racial and/or ethnic groups in science. We actively worked to promote sex and gender balance in our author group. We actively worked to promote inclusion of historically underrepresented racial and/or ethnic groups in science in our author group. While citing references scientifically relevant for this work, we also actively worked to promote sex and gender balance in our reference list. While citing references scientifically relevant for this work, we also actively worked to promote inclusion of historically underrepresented racial and/or ethnic groups in science in our reference list. One or more of the authors of this paper self-identifies as a member of one or more historically underrepresented sexual and/or gender groups in science. One or more of the authors of this paper self-identifies as living with a disability.

## Plain language summary

Starting college is a major life transition for young adults, making it an important period for promoting healthy behaviors. In the Lived Experiences Measured Rings Study (LEMURS), we found that individual characteristics like gender, personality, mental health diagnoses, and past traumatic experiences predicted level of anxiety in a first-year college cohort during their first semester at a public university in the northeastern United States. The study also found that several weekly factors strongly predicted anxiety. Specifically, each additional hour of reported sleep was associated with a 0.589-point decrease in anxiety, while each additional hour of objectively recorded sleep reduced anxiety by 0.491 points. Poor sleep quality and low sleep satisfaction were linked to increases in anxiety by 1.176 and 1.348 points, respectively, and the presence of academic stressors, such as papers or exams, increased anxiety by 1.352 points. These results highlight the critical role of both sleep and academic stress in shaping students’ mental health during their transition to college.

Anxiety disorders are a leading cause of disability globally and affect 1 out of every 30 people in their lifetime.^1^ Symptoms of generalized anxiety disorder (GAD) often first present in early adulthood (18-24 years of age).^2^ GAD and other common mental health disorders, such as depression, are internalizing disorders that lead to emotional distress in response to life stressors and previous traumatic events.^3^^,^^4^ Young adults in the United States suffer from anxiety disorders 2 times more often than the rest of the adult population, and the onset of these disorders has been associated with poor academic outcomes, suicide risk, drug and alcohol misuse, and risky behaviors.^5^ Psychological distress and anxiety symptoms during young adulthood are also linked to worse career outcomes, adverse mental health outcomes, and poor well-being later in life.^6^

In particular, college has been linked to common mental health disorders such as anxiety, because it is a period of emerging adulthood—the transition from adolescence to adulthood—which includes changes in physical context, social relationships, and increased independence.^5^ In a recent study, anxiety increased from the summer before college through the first year of college.^7^ In a global study of college students, more than one-third reported a common mental health disorder during their first year, and were widely distributed across demographic characteristics.^8^ The first 2 years of college have been associated with worsening psychological functioning, poor social adjustment, and decreased coping skills.^9^ Suicide is among the leading causes of death among college students, and an estimated 11% of young adults in the United States report having serious thoughts of suicide.^10^ Furthermore, those with a diagnosis of a mental health condition and current symptoms of depression and/or anxiety have increased odds of suicidal ideation as well as increased odds of planning and attempts.^11^

Individual traits and exposure to stressful life events are associated with an increased risk of developing an anxiety disorder.^12^^,^^13^ Longitudinal studies have shown that being female and having a lower socio-economic status increases the risk of developing anxiety.^9^^,^^14^ Also, certain personality traits have been linked to anxiety and differences in stress responses and recovery, especially during college.^15^, ^16^, ^17^ Many young adults begin their college experience with a mental health diagnosis associated with worsening mental health during this period.^18^ In a recent study, students who had depression and anxiety at the beginning of college were less likely to recover from anxiety by the end of their first year.^19^ However, most estimates of the mental health burden for young adults are cross-sectional, and much less is known about the course of anxiety symptoms during transitional periods. Recent longitudinal studies have also shown that anxiety symptoms significantly change from 1 month to another.^19^ Anxiety symptoms may fluctuate and therefore opportunities for time-sensitive interventions may be missed.

College is also a period marked by insufficient sleep and irregular sleep patterns, which influence physical and mental health.^20^ A recent multi-university study found that more than 60% of college students met the criteria for poor sleep, and that mental health symptoms were associated with decreased sleep quality.^21^ Evidence suggests that stress from significant events in early life is a risk factor for poor sleep,^22^ and higher levels of distress during a stressful transition is associated with poorer sleep quality in university students.^6^ There is a growing body of literature showing that poor sleep quality is associated with decreased academic performance,^23^ and that total sleep time is an important predictor for success in college,^24^ daily measures of mental health,^25^^,^^26^ and even health outcomes later in life.^27^^,^^28^

Although self-assessment measures are effective ways to assess the severity of and to distinguish between common mental health concerns, including the Generalized Anxiety Disorder Questionnaire (GAD-7), which has been validated in young adult populations,^29^ survey-based methods that require active engagement can pose a burden on participants, and these studies often suffer from attrition.^30^ Recent work using biometric wearables has provided insight into differences in sleep for people with mental health diagnoses compared to those without, and has shown promise in predicting stress from these measures.^31^^,^^32^ Identifying key predictors for elevated anxiety during the first year of college and assessing the concordance of self-reported and passively collected information on sleep may help to target resources to better support student well-being during this time.

This study builds an individualized model of mental well-being during this important period, with a focus on factors that influence the primary outcome of anxiety. In the present longitudinal study, we investigated whether individual traits, previous traumatic exposure, and behavioral factors could be used to predict self-reported measures of anxiety in first-year college students. We hypothesized the following: (1) demographic characteristics such as a gender identity, first-generation college status, and racial minority status would be associated with elevated anxiety; (2) a previous mental health diagnosis and previous exposures to traumatic events would be associated with elevated anxiety; (3) personality traits, such as high extraversion, high agreeableness, high openness, and low neuroticism, would be associated with resilience to stressors and would be associated with decreased anxiety; (4) improved sleep measures would be associated with lower anxiety and improved trajectories of anxiety; and (5) acute stressors including papers and examinations would increase anxiety symptoms over the course of the semester. Given the health implications of prolonged anxiety, understanding the factors that influence the trajectories of anxiety in young adults offers the potential for targeted, preventive interventions.

## Method

### Study Design and Sample

This study protocol was reviewed and approved by the University of Vermont Institutional Review Board (UVM IRB). Participants were recruited during orientation during the fall semester of their first year of university, through student mailing lists and in-person events that included tabling at the student center from August 21, 2022, to October 15, 2022. A total of 605 undergraduate students were eligible to participate. Inclusion criteria was being a first-year student between the ages of 18 and 24 years, being full-time (enrolled in at least 12 credits), and owning a smartphone. Interested participants who did not meet these criteria were excluded and not enrolled. After the eligibility screening, participants were invited to attend a lecture that provided in-depth information about the study. Participants were required to complete a comprehension assessment with completely correct answers before being able to provide written informed consent through REDCap, an online application that is compliant with the Health Insurance Portability and Accountability Act (HIPPAA).

**Figure 1:**
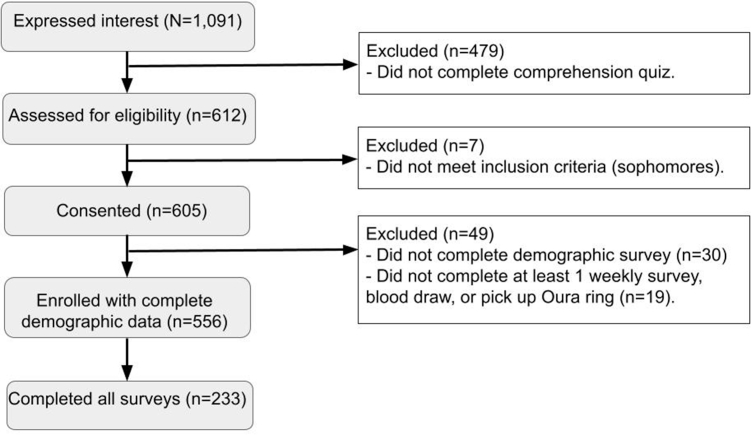
Study Flow Diagram ***Note:****Participants through study screening, consent, and completion.*

Following consent, enrolled participants completed a baseline survey, and weekly surveys were administered through REDCap between October 21, 2022, and December 12, 2022. Participants were enrolled over 2 weeks (weeks 1 and 2 below), which served as the baseline measurement for participants. After enrollment, each participant was assigned a study identifier. Participants were asked to complete a basic blood panel at the Clinical Research Center and a health history survey. Participants then attended an in-person event to complete sizing for their Oura Ring, a consumer wearable. The Oura Ring automatically collected body response data during sleep and daily activity. Those data were uploaded to Oura Cloud via the Oura mobile app and were accessed using the Oura mobile app or Oura on the Web. When participants were enrolled in Oura Teams, all identifying information was removed and manually coded with their unique study identifier. Oura Teams’ data were only accessed by those approved by the UVM IRB. Responses to questions from electronic questionnaires were stored in REDCap. All data collected by this study were stored on a secure virtual machine on a drive that restricted access to those approved by the UVM IRB. The study followed the Strengthening of Reporting of Observational Studies in Epidemiology (STROBE) reporting guidelines for cohort studies. A flow diagram shows the reasons for exclusion at each stage (Figure 1).

### Measures

#### Anxiety

Symptomatology of anxiety was measured using the Generalized Anxiety Disorder Questionnaire—7 (GAD-7).^29^ The GAD-7 is a 7-item self-report scale in which anxiety-defining items are rated on a 4-point Likert scale (from 0 = not at all to 3 = nearly every day), resulting in a composite score between 0 and 21. GAD-7 items describe salient features of anxiety (eg, feeling on edge and worrying too much about various things) experienced within the past 2 weeks. The GAD-7 has good reliability in both clinical and population-based samples when validated against assessments by mental health professionals, which are considered the gold standard.^33^^,^^34^ The Cronbach alpha value was 0.90 for GAD-7 scores, revealing excellent internal consistency for this measure.

#### Demographic Characteristics

We collected self-reported information on gender, race, ethnicity, and first-generation college status of all participants at the time of enrollment. We included 3 gender categories in our analysis: male, female, and gender minority. The category of gender minority included participants who identify as nonbinary, transgender, genderfluid, and agender. There was minimal variation in reported age, and therefore this variable was excluded from analysis.

#### Personality Traits

Personality traits describe the self-efficacy with which individuals appraise and cope with life challenges, which have been used to understand stress response and coping behaviors.^35^ The Ten Item Personality Inventory (TIPI) is a 10-item measure that identifies dimensions of the “Big Five,” widely recognized as a personality trait model.^36^ Each trait is represented by 2 items, 1 item stated in a way that represents the positive pole of the dimension and the other item stated in a way that represents the negative pole. Each item was scored from 1 (strongly disagree) to 7 (strongly agree). Five of the items are reverse scored. The 2 scores, the negative and positive poles, are then averaged for a composite score.

#### Mental Health

Participants were asked: “Do you have a history of an underlying mental health condition?” If participants selected “yes,” they were prompted to select from the following list of common mental health conditions: anxiety, depression, attention-deficit/hyperactivity disorder (ADHD), alcoholism, psychosis, delusions, anorexia or bulimia, post-traumatic stress disorder (PTSD), obsessive-compulsive disorder (OCD), bipolar disorder (BPD), panic attack disorder, or an emotional disorder. We created a binary variable for anxiety diagnosis to indicate an underlying anxiety disorder, but sample frequencies of all mental health diagnoses are included in Table 1.

**Table 1:** Baseline Demographic Data, Weekly Surveys, and Anxiety Measures for Participants

Baseline demographics (categorical)	% (n)
Gender	
Female	66.01 (367)
Gender minority	6.29 (35)
Male	27.69 (154)
Race	
Non-White	11.87 (66)
American Indian	0.36 (2)
Asian-Pacific	5.39 (30)
Biracial/multi-race	5.39 (30)
Black	0.72 (4)
White	88.13 (490)
Ethnicity	
Hispanic	5.22 (29)
Non-Hispanic	94.78 (527)
First-generation	
First in family to college	8.45 (47)
Not first in family to college	91.55 (509)
Life Events Checklist score	
0	28.96 (161)
1	31.65 (176)
≥2	39.39 (219)
Mental health history	
Mental health diagnosis (any)	44.06 (245)
Anxiety disorder	39.21 (218)
Depression	31.12 (173)
ADHD	10.97 (61)
Anorexia or bulimia	7.37 (41)
OCD	6.29 (35)
PTSD	3.60 (20)
Panic	3.78 (21)
Emotional disorder	1.26 (7)
Bipolar	1.08 (6)
Alcoholism	0.72 (4)
Delusions	0.18 (1)
Psychosis	0.18 (1)

**Baseline traits (Likert scale)**	**Mean (SD)**

Personality trait	
Openness	2.49 (0.91)
Conscientiousness	2.27 (1.07)
Extraversion	3.70 (1.43)
Agreeableness	3.01 (1.09)
Neuroticism	3.69 (1.24)


Weekly behaviors and events	Mean (SD)
Sleep	
Sleep time	
Reported nightly sleep duration, h, survey	7.12 (1.05)
Recorded nightly total sleep time, h, Oura Ring	7.39 (0.83)

	**% Responses**

Sleep quality	
Fairly or very good	67.27
Poor sleep	32.79
Sleep satisfaction	
Too little sleep	45.73
Adequate or ideal sleep	54.27
Stressors	
Stressful event	
Yes	37.64
No	62.36
Paper or examination	
Yes	24.22
No	75.78

**Outcome measures**	**Mean (SD)**

Sample level	
GAD-7	6.50 (5.33)
Individual level	
GAD-7	6.60 (4.65)

Note: Descriptive statistics for enrolled participants with complete demographic information who completed at least 1 weekly survey (N = 556) and had corresponding Oura Ring data. ADHD = attention-deficit/hyperactivity disorder; GAD-7 = Generalized Anxiety Disorder Questionnaire—7; OCD = obsessive-compulsive disorder; PTSD = post-traumatic stress disorder.

#### Traumatic Exposures

The Life Events Checklist (LEC) assesses the occurrence of major life events that a person has experienced, witnessed, or learned about happening to someone close to them that are potentially traumatic.^37^ In our sample, the LEC was collected at baseline for all participants. Although there are multiple LEC scoring approaches, we totaled items 1 to 16 that were endorsed as experienced only, in line with previous researchers.^37^^,^^38^ We then created an ordinal variable for no exposure (0), a single exposure (1), and multiple (≥2) exposures to traumatic life events.

#### Acute Stressors

Participants were asked: “Was there an event or events this week that were particularly stressful?” Then participants were asked to characterize the nature of the stressful event (ie, social, romantic, familial, financial, academic, physical, or mental). Participants who indicated that they had an academic stressor were assigned a value of 1; those who did not were assigned a value of 0. Participants were also asked whether they had a test or project due in the last week. We created a binary variable to indicate whether the participant had a test or project due in the previous week.

#### Sleep Assessment

Participants were asked 3 questions to assess their perceived sleep quantity, quality, and satisfaction. For reported sleep duration, they were asked: “On average how many hours of sleep did you get per night in the past week?” which required numerical input. For perceived sleep quality, they were asked: “Over the past week, how would you rate your sleep quality?” Responses were on a Likert scale ranging from very bad (1) to very good (4). We created a binary variable for sleep quality. Participants who responded that their sleep quality was quite bad and very bad were rated as having poor sleep quality. Finally, participants were asked about their sleep satisfaction: “This week, do you feel like you got…?” Responses were on a Likert scale, from too little sleep (1) to too much sleep (4). We created binary variables for sleep satisfaction. Participants who responded that they had too little sleep were considered to have low sleep satisfaction.

#### Recorded Sleep Duration

To objectively quantify sleep, participants wore a consumer wearable, the Oura Ring (Oura Health, Ltd), which uses a combination of accelerometer data, heart rate, and pulse wave amplitude variability with machine learning models to calculate sleep duration.^39^ The Oura Ring has a high association with polysomnography for measuring total sleep time.^39^ For each night, we used information recorded by the Oura Ring for the sleep period with the longest duration (ie, naps were not included). To compare the weekly survey measures to the daily Oura sleep data, we averaged the daily sleep measurements at a weekly level. For every user, we considered weeks in which there were at least 3 days for which sleep data were recorded. From the raw Oura data, 94.5% of user–week combinations (3,530 of 3,735) had 3 or more Oura data points per week.

### Data analysis

#### Statistical Analysis

We used longitudinal mixed-effects models due to the nested structure of panel data. These models can handle missing and unbalanced data more effectively than traditional regression methods and account for within-individual and between-individual variability.^40^^,^^41^ Covariates included gender, race, and first-generation college status. Time-invariant predictors included personality traits, having a previous diagnosis of an anxiety disorder, and previous exposure to potentially traumatic life events (LEC). Time-varying predictors were week of the semester, academic stressors (ie, tests and projects), perceived sleep quality, perceived sleep satisfaction, perceived nightly sleep duration, and average nightly hours of sleep estimated from the Oura Ring. Supplemental tables for correlation between predictors and outcome variables are included (Tables S1-S3, available online)

We explored multiple model forms, but our final model was a mixed-effects linear regression model with random effects for intercept and the slope of week in the semester. The final model form was as follows: *GAD*_*ij*_ = (*β*_00_ + *β*_*01, 02,... 0S*_ Time Invariant Predictors_*i*_ +*u*_0i_) + (*β*_10_ week_ij_ +*u*_1i_) +  (*β*_20,30…_ s Time VaryingPredictors_*ij*_)+ *ε*_*ij*_ where S represents the set of included predictors (Table S4, available online). Additional predictors were added to the model to evaluate specific hypotheses. Missing data were handled with maximum likelihood estimation as part of the mixed-effect modeling process. Supplemental tables for analyses are included online for the reader’s information (Tables S5-S7, available online).

#### Power Analysis

A sample size of 600 individuals is sufficient to detect group differences at the size of *d <* 0.1. It was anticipated that the effect size for anxiety would be *d =* 0.3, so with a 20% attrition rate (end sample N = 480), we expected that the sample size would be sufficient to detect these differences.

#### Sensitivity Analysis

Type I error was controlled using the Bonferroni correction with the false discovery rate set to 5%.

## Results

### Participants

There were 1,091 individuals who responded to outreach and expressed interest in the study. Of these, 56.1% (612 of 1,091) completed the screening questionnaire and comprehension quiz. In all, 98.9% (605 of 612) were confirmed to be eligible, consented to participate, and completed the study enrollment procedures, which included a baseline survey, a clinical assessment, and getting fit for an Oura Ring (Figure 1). The final analysis included data from 91.9% (556 of 605) of consented participants who had complete demographic data, baseline data, and did not withdraw from the study.

We observed a high level of compliance and survey response from participants who were eligible and enrolled in the study. Of those with complete demographic information, participants completed an average of 5.68 surveys (SD = 1.57) of a possible 7 responses for a total of 3,159 survey responses. Of the participants, 94.4% (525 of 556) completed 3 or more surveys, and 41.9% (233 of 556) completed all 7 weekly surveys. Descriptive statistics of the reduced samples had distributions similar to those of an inclusive sample (Table S8, available online).

In our primary analyses, we included participants who completed at least 1 of 7 possible weekly surveys and had complete demographic data (N = 556). The majority identified as the female gender (n = 367) and White (n = 490) (Table 1). Consistent with national estimates, 6% (n = 35) of our sample self-identified as a gender minority, a gender different from that assigned sex at birth (ie, non-binary, genderfluid, transgender, and agender).

### Trajectories of Anxiety

**Figure 2:**
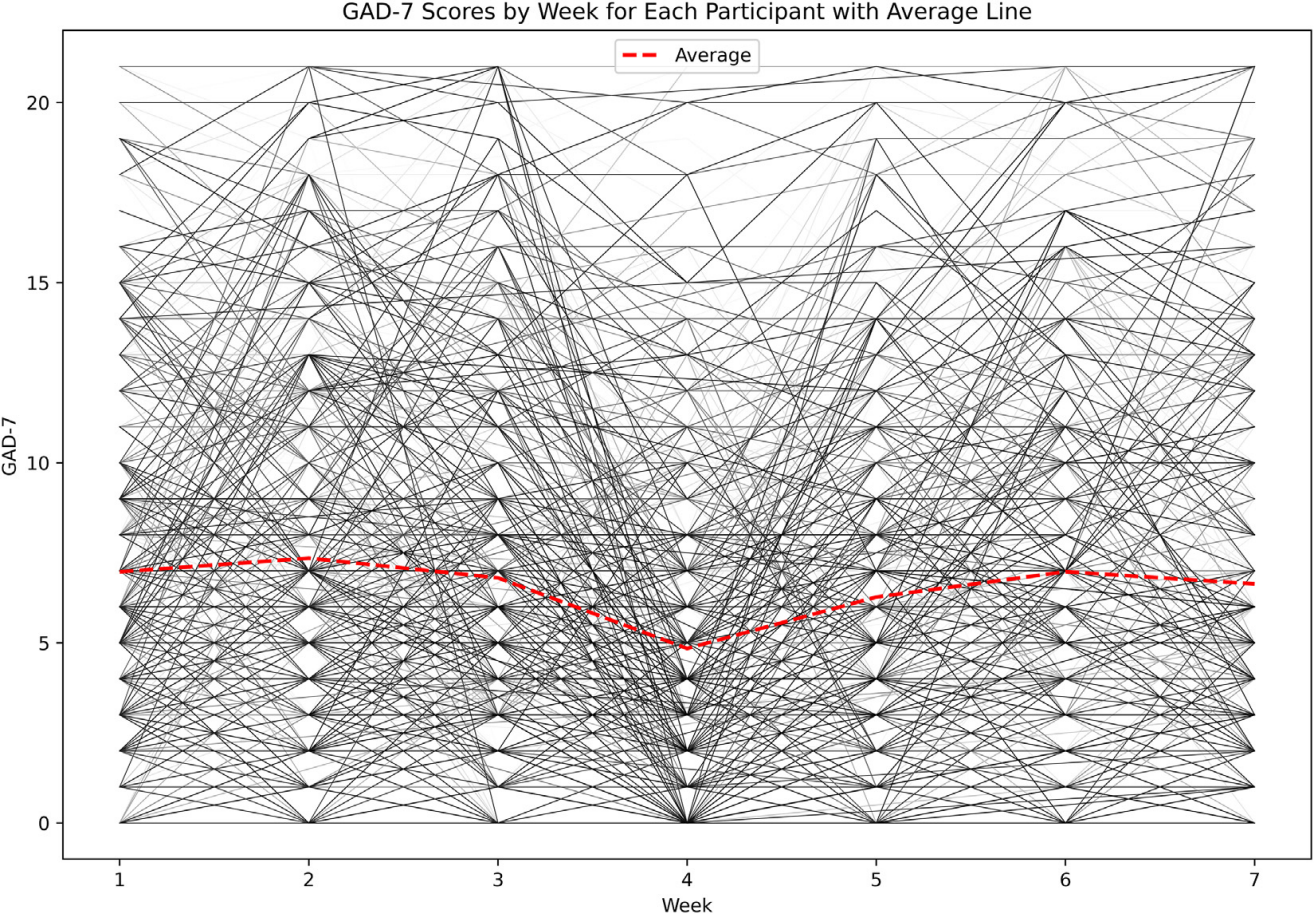
Trajectories of Anxiety Across the First Semester of College ***Note:****Red line depicts the average GAD-7 score of the sample. Lines represent individual regression lines by participant. GAD-7 = Generalized Anxiety Disorder Questionnaire—7*.

The average GAD-7 score was 6.50 (SD = 5.33) for participants over the duration of the study. Week 3 had the highest average GAD-7 score of the study period (mean = 7.30, SD = 5.36), and week 5 had the lowest average GAD-7 score (mean = 4.83, SD = 4.72), which corresponded to Thanksgiving break. As far as fluctuations in anxiety, the average change in GAD-7 scores between weeks was −0.05 (SD = 4.02). The average variance in a participant’s GAD-7 score over the study was 6.38 (SD = 4.16), showing that participants had varying levels of anxiety throughout the course of the study (Figure 2).

**Figure 3:**
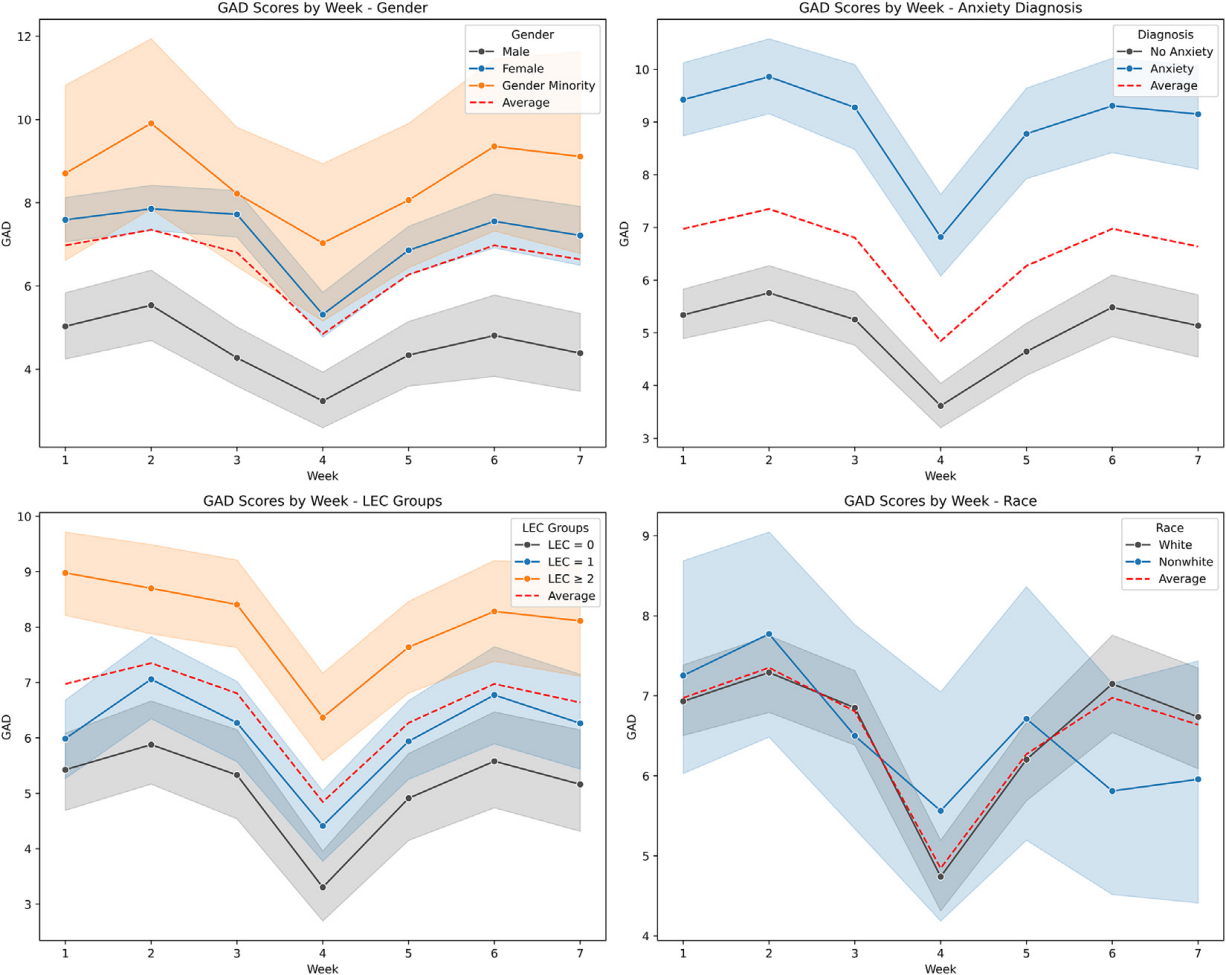
Weekly Distribution of Anxiety, by Baseline Demographic Predictors ***Note:****Dashed red line depicts the average anxiety trajectory of the sample for all figures. (a) Weekly average GAD-7 score by gender identity; (b) weekly average GAD-7 scores for those with and without a history of anxiety disorder diagnosis; (c) weekly average GAD-7 scores for those with history of potentially traumatic life events (LEC = 0, no events; LEC = 1, one event; LEC ≥2, at least 2 events); d) weekly average GAD-7 scores by first-generation college status. GAD-7 = Generalized Anxiety Disorder Questionnaire—7; LEC = Life Events Checklist.*

The mixed-effects model suggested that anxiety significantly decreased over the semester (b = 0.092, *p* = .007). Female gender and gender minority status were significantly associated with higher GAD-7 scores compared to male gender (b = 2.536, *p* < .001 and b = 4.001, *p* < .001, respectively) (Table 2, Figure 3a). Other demographic variables (eg, first-generation status) were not significantly associated with anxiety trajectories (Table 2, Figure 3d).

**Table 2:** Parameter Estimates for Models Predicting Anxiety

Covariate	GAD-7		
	Coefficient	95% CI	Adjusted^a^*p*
Gender			
Female	2.536	1.708, 3.363	**.000**
Gender minority	4.001	2.394, 5.619	**.000**
Race			
Non-White	−0.199	−1.374, 0.975	.739
Ethnicity			
Hispanic	−0.222	−1.929, 1.486	.799
First-generation			
First in family to college	0.311	−1.048, 1.670	.654
**Time invariant predictors**			
Life Events Checklist			
1	0.994	0.055, 1.934	.039
≥2	2.938	2.044, 3.833	**.000**
Mental health history			
Mental health diagnosis (any)	3.922	3.229, 4.614	**.000**
Anxiety diagnosis	3.962	3.257, 4.667	**.000**
Personality traits			
Openness	0.210	−0.210, 0.630	.326
Conscientiousness	0.245	−0.179, 0.669	.256
Extraversion	0.539	0.280, 0.799	**.000**
Agreeableness	0.065	−0.285, 0.415	.716
Neuroticism	1.881	1.617, 2.145	**.000**
**Time-varying predictors**			
Sleep			
Reported nightly sleep duration, h, survey	−0.589	−0.712, -0.467	**.000**
Recorded nightly total sleep time, h, Oura Ring	−0.491	−0.666, -0.314	**.000**
Poor sleep quality	1.176	0.916, 1.436	**.000**
Low sleep satisfaction	1.348	1.098, 1.598	**.000**
Stressors			
Stressful event	2.168	1.930, 2.408	**.000**
Paper or examination	1.352	1.101, 1.594	**.000**

Note: Model form for all models: GAD_ij_ = (β_00_ + β_01, 02,... 0S_ Time Invariant Predictors_i_ +u_0i_) + (β_10_ week_ij_ +u_1i_) +  (β_20,30…_ s Time VaryingPredictors_ij_)+ ε_ij_. GAD-7 = Generalized Anxiety Disorder Questionnaire—7.

a Adjusted using the Bonferroni correction with the false discovery rate set to 5%, which with 1 test is 0.005.

Participants with an underlying anxiety disorder and those with repeated exposure to potentially traumatic life events (LEC ≥2) had significantly higher average GAD-7 scores (b = 3.962, *p* < .001 [Table 2, Figure 3b] and b = 2.938, *p* < .001, respectively [Table 2, Figure 3c]). A relationship between GAD-7 scores and the personality traits of extraversion and neuroticism was observed across covariate-adjusted mixed-effects regression models. For every 1-unit increase in the extraversion trait, there was an estimated increase in GAD-7 score (b = 0.507, *p* < .001) (Table 2, Figure S2c, available online). Conversely, for every 1-unit increase in the neuroticism trait, there was a 1.753 estimated increase in GAD-7 score (*p* < .001) (Table 2, Figure S2d, available online).

Weekly stressors were associated with GAD-7 scores as well. Self-reported average nightly hours and average nightly hours of sleep recorded from biometric devices were significantly negatively associated with GAD-7 scores (b = −0.589, *p* <.001 [Table 2, Figure S3a, available online] and b = −0.491, *p* < .001, respectively [Table 2, Figure S3b, available online]). Self-reported poor sleep quality was significantly associated with GAD-7 scores (b = 1.176, *p* < .001) (Table 2, Figure S3c, available online). Self-reported low sleep satisfaction was associated with GAD-7 scores (b = 1.348, *p* < .001) (Table 2, Figure S3d, available online). Having a paper or an examination on a given week was associated with a significant increase in GAD-7 scores (b = 1.352, *p* < .001) (Table 2).

## Discussion

The transition to university is a time marked by elevated levels of psychological distress.^42^ Students are often living independently for the first time, have new academic challenges, and with these newfound freedoms are navigating risky health behaviors and new social relationships. This study examined student anxiety during the transition to college, and how individual traits, prior exposures to traumatic life events, weekly stressors, and sleep influenced anxiety trajectories. We also examined whether self-reported sleep assessments correspond to wearable biometric data estimates in this period of potentially heightened anxiety. Although other studies have looked at the development of anxiety over the first year of college, trajectories in anxiety symptoms have not been well described.^7^^,^^8^^,^^19^^,^^43^

We had 6 main findings. First, there were significant differences in anxiety symptoms by gender identity. Second, a previous mental health diagnosis and traumatic exposures were strong predictors of anxiety symptoms. Third, the personality traits of extraversion and neuroticism were significant predictors of increased anxiety symptoms. Fourth, self-reported sleep duration, poor sleep quality, and low sleep satisfaction were significant predictors of anxiety symptoms. Fifth, estimates of an average nightly sleep duration from a consumer wearable was also a significant predictor of anxiety in covariate-adjusted, corrected models. Sixth, academic stressors were significant predictors of anxiety symptoms during the semester.

We observed a high level of compliance and survey response from participants who were eligible and enrolled in the study compared to other studies.^44^ The prevalence of anxiety in our sample is consistent with previous samples of university students.^45^ Variability in anxiety was clear across gender categories and is consistent with previous studies showing higher anxiety scores in non-male participants.^14^ Previous work indicates that university students who have experienced trauma earlier in life struggle more with the adjustment to university compared to students with no history of trauma.^12^ There are unmeasured behavioral changes that may influence mental well-being. Previous studies have found that familial support, living in urban areas, and a steady family income were protective against anxiety in college students.^14^^,^^46^

Mental health, sleep, stress response, and anxiety symptoms are interrelated. Our study illustrated this in models in which acute stressors and sleep measures were significant predictors of weekly trajectories in anxiety. In a large meta-analysis, it was demonstrated that there was disrupted sleep continuity with significant reduction of total sleep time for patients with mental health disorders.^47^ Generalized anxiety disorder (GAD) is the most prevalent symptomatology for individuals complaining of poor-quality sleep and insomnia.^48^ An estimated 60% to 70% of patients with anxiety disorders have trouble initiating and maintaining sleep, pointing to interactions between sleep and mental health.^47^^,^^49^ Most university students report sleep disturbances, and test anxiety is correlated with disrupted sleep and poor sleep quality.^20^ Anxiety associated with tests and projects has been well documented across student populations, and sleep has a significant impact on academic success.^24^^,^^50^

The addition of biometric data to our analysis was used as an objective measure to validate self-reported measures and perceptions of sleep. The use of wearable devices in college cohort studies has shown promise in identifying changes in mental health.^51^ A study using wearable devices in college students showed that better quality, longer duration, and greater consistency of sleep was correlated with better grades.^52^ However, this study used only sleep duration estimates from the Oura Ring’s longest nightly sleep period, which was averaged by week. It will be important for future studies to evaluate additional sleep variables, such as daytime naps, associated with mental health in college students.

The study results reinforce that trajectories of anxiety during the transition to college are the product of patterns of multiple individual traits and behaviors that are shaped by the stressors that students experience. This has important implications for intervention. During the transition to college, well-being requires addressing factors that contribute to stressors that are non-uniformly distributed among students. Many studies have pointed to in-person and online programs that target self-compassion in higher education settings, which have been efficacious at decreasing the effect of stressors on mental health in college students.^7^^,^^53^^,^^54^ To reach the large college student population globally, more research on the frequency, timing, and dosage of sessions that are effective at increasing protective factors such as self-compassion and decreasing anxiety is warranted. Settings of higher education can bring together administrators and health care professionals to invest in programs that support students during this transition period.

Although self-reported GAD-7 measures are not analogous to a confirmed anxiety diagnosis, they provide insight into the anxiety trajectories for young adults in their first year of college, as well as the factors that influence these trajectories. However, the use of self-report outcome measures, covariates, and predictors could lead to an increased risk of social desirability response bias. There is also the potential for repeated administration of the self-report measure to lead to decreased reporting of anxiety. Inaccurate estimation of the proportion of participants with a previous mental health diagnosis and exposure to traumatic experiences could be present if the event occurred far in the past. Our sample was predominantly female, White, non-Hispanic, and composed of participants who were not first-generation college students, and there are also potential limitations in the applicability of these findings to more diverse populations. Gender imbalance is common in survey-based research and is consistent with current undergraduate student populations, but future work would benefit from inclusion of additional cohorts to assess the influence of these traits and applicability of these results to a broader population. Also, we do not have anxiety, stress exposure, or sleep data from before participants moved to college, and we cannot assess the influence of beginning college on these measures, which may have shifted at the start of college.

Expanding this window of analysis may identify whether the link between anxiety, external stressors, and self-reported and objective sleep measures holds prior to the beginning of college and throughout the college experience. Compared to previous studies in first-year college students,^20^^,^^21^^,^^24^ this cohort got more objective sleep, pointing to potential differences in this population from other college-aged groups. Finally, potential confounders also exist in our data. In our analysis, we did not account for some factors that may influence anxiety and sleep measures, including psychotropic medications, sleep disorders, physical activity, or the use of substances (eg, caffeine, marijuana, and alcohol). Despite these limitations, our study demonstrates the importance of higher temporal granularity in longitudinal studies on anxiety in young adults.

With an increased focus of universities on the mental health and well-being of students, the advent and success of mobile assessments of anxiety, and the growing evidence for single time-point interventions, the utility of following young adults through a potentially turbulent period of their life holds promise for reducing morbidity and mortality in this age group and supporting long-term health through individualized interventions. Future work will investigate the potential of scalable interventions to improve mental health in this population by monitoring mental health and offering evidence-based behavioral interventions.^55^ This study provides a framework for individual-centered programs. Understanding the dynamic processes that cause students to have different anxiety trajectories is essential for successfully choosing and targeting interventions in this critical developmental period.

In their first semester of college, young adults experience a major life transition change associated with anxiety. Many students arrive at college with a history of mental health disorders, exposure to traumatic life events, and health behavior patterns that are associated with higher levels of anxiety. The personality traits of extraversion and neuroticism also played a role in the presence of anxiety symptoms, which may be important for personalizing interventions. As expected, weekly stressors and reduced sleep measures were significant predictors of anxiety trajectories. The present findings call for more research following young adults who are at elevated risk for increased anxiety during transition periods. The first year of college is an opportunity to engage young adults in health behavior interventions, given the implications for increased morbidity and mortality associated with poor mental health in this population.

## CRediT authorship contribution statement

**Laura S.P. Bloomfield:** Writing – original draft, Visualization, Methodology, Investigation, Funding acquisition, Formal analysis, Data curation, Conceptualization. **Mikaela Irene Fudolig:** Writing – review & editing. **Julia N. Kim:** Project administration. **Jordan** **Llorin:** Project administration. **Juniper Lovato:** Writing – review & editing. **Ellen W. McGinnis:** Writing – review & editing, Investigation. **Ryan S. McGinnis:** Writing – review & editing, Investigation. **Matthew Price:** Writing – review & editing, Supervision, Methodology, Investigation, Conceptualization. **Taylor H. Ricketts:** Writing – review & editing. **Peter Sheridan Dodds:** Funding acquisition. **Kathryn Stanton:** Project administration. **Christopher M. Danforth:** Writing – review & editing, Supervision, Resources, Methodology, Investigation, Funding acquisition.
