# Supplementary material for: Predictors of Anxiety Trajectories in Cohort of First-Year College Students
Source: JAACAP Open. 2024 Oct 18;3(3):805–16. doi: 10.1016/j.jaacop.2024.08.004 (PMC12414336; doi:10.1016/j.jaacop.2024.08.004)
Supplement: Supplementary Figures and Tables [file mmc1.docx]

**Figure S1: Anxiety Distribution for Cohort over Study**

**Note:** a) Distribution of GAD-7 scores during study and b) Imputed values for outcome variables. Distribution of GAD-7 vs Imputed values for missing weeks (N=3,159). GAD-7 = Generalized Anxiety Disorder Questionnaire; GAD-meanimp = Missing values are replaced by the mean value within each participant; GAD-medianimp = Missing values are replaced by median of the observed values within each participant; GAD-locfimp = Missing values are replaced by last observed value for that variable for each participant; GAD-knnimp = Missing values are replaced by values of nearest neighbors for that variable for each participant

**
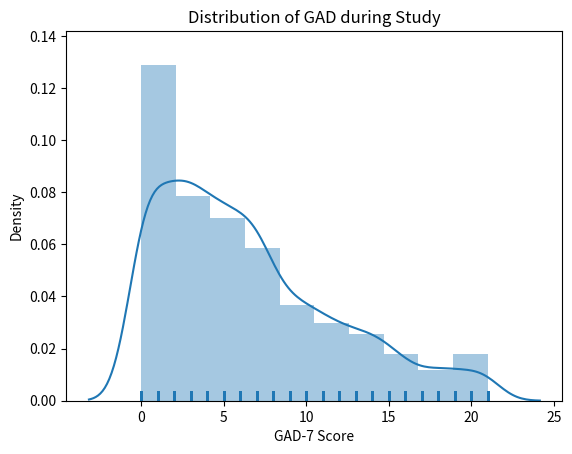
**
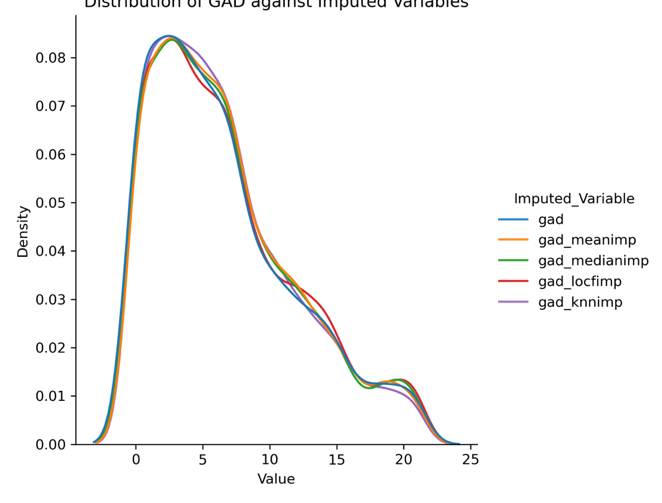


**Figure S2.** **Relationship Between Anxiety and Personality Traits**

**Note:** S2a) Scatterplot of TIPI-Openness and GAD-7 score, S2b) Scatterplot of TIPI-Agreeableness and GAD-7 score, S2c) Scatterplot of TIPI-Extraversion and GAD-7 score, S2d) Scatterplot of TIPI-Neuroticism and GAD-7 score. GAD-7 = Generalized Anxiety Disorder Questionnaire; TIPI = Ten Item Personality Inventory.


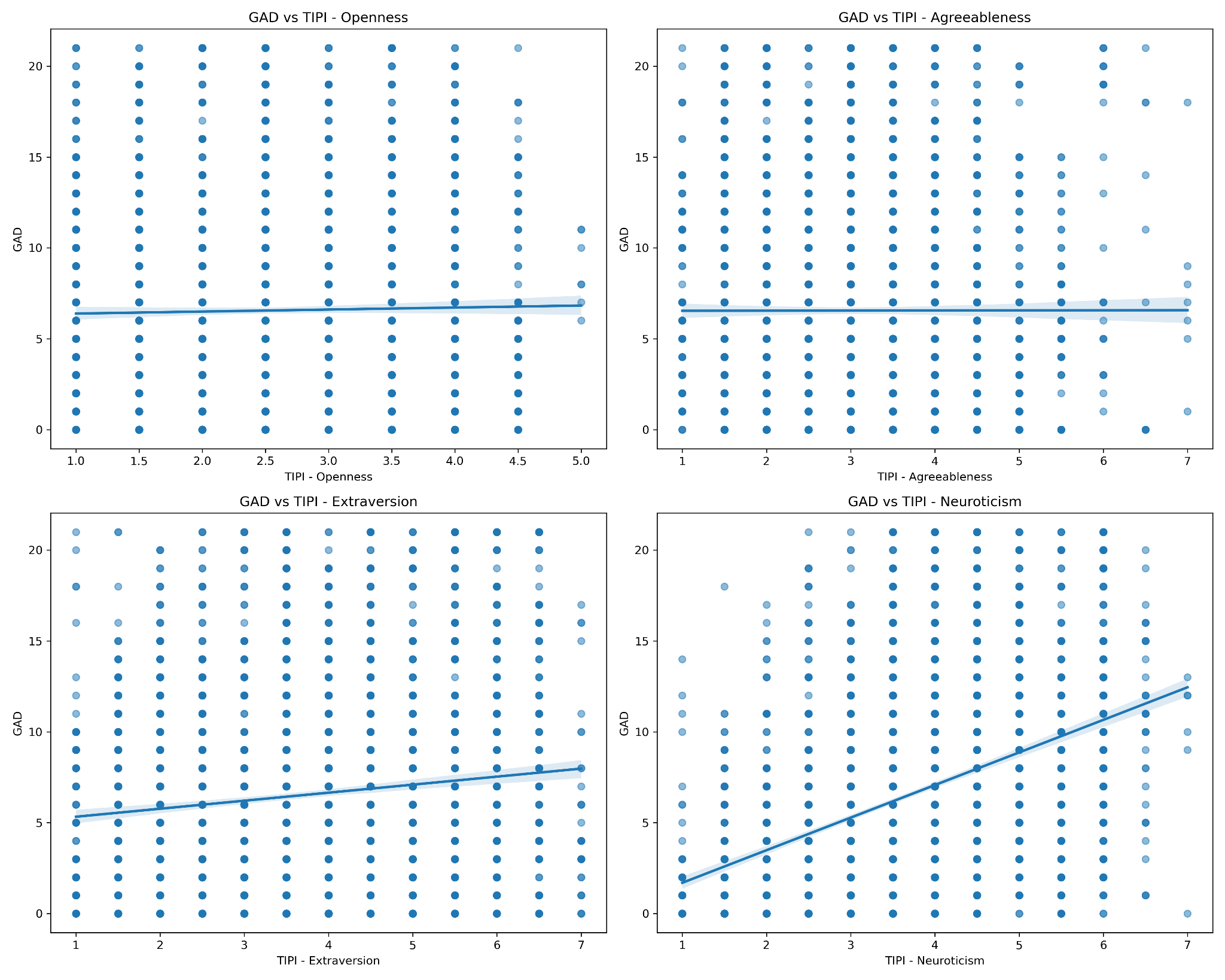


**Figure S3.** **Relationship Between Anxiety and Sleep Measures.**

**Note:** 3a) Histogram and fit line between hours of reported sleep and GAD-7 score, 3b) Histogram and fit line between average hours of recorded sleep from Oura Ring and GAD-7 score, 3c) Boxplot of reported sleep quality (from very good (4) to very bad (1= very bad, 2 = fairly poor , 3 = fairly good, 4 = very good) and GAD-7 score, 3d) Boxplot of reported sleep satisfaction (1= too little sleep; 2 = adequate sleep, 3 = ideal, 4= too much sleep) and GAD-7 score. GAD-7 = Generalized Anxiety Disorder Questionnaire.

**
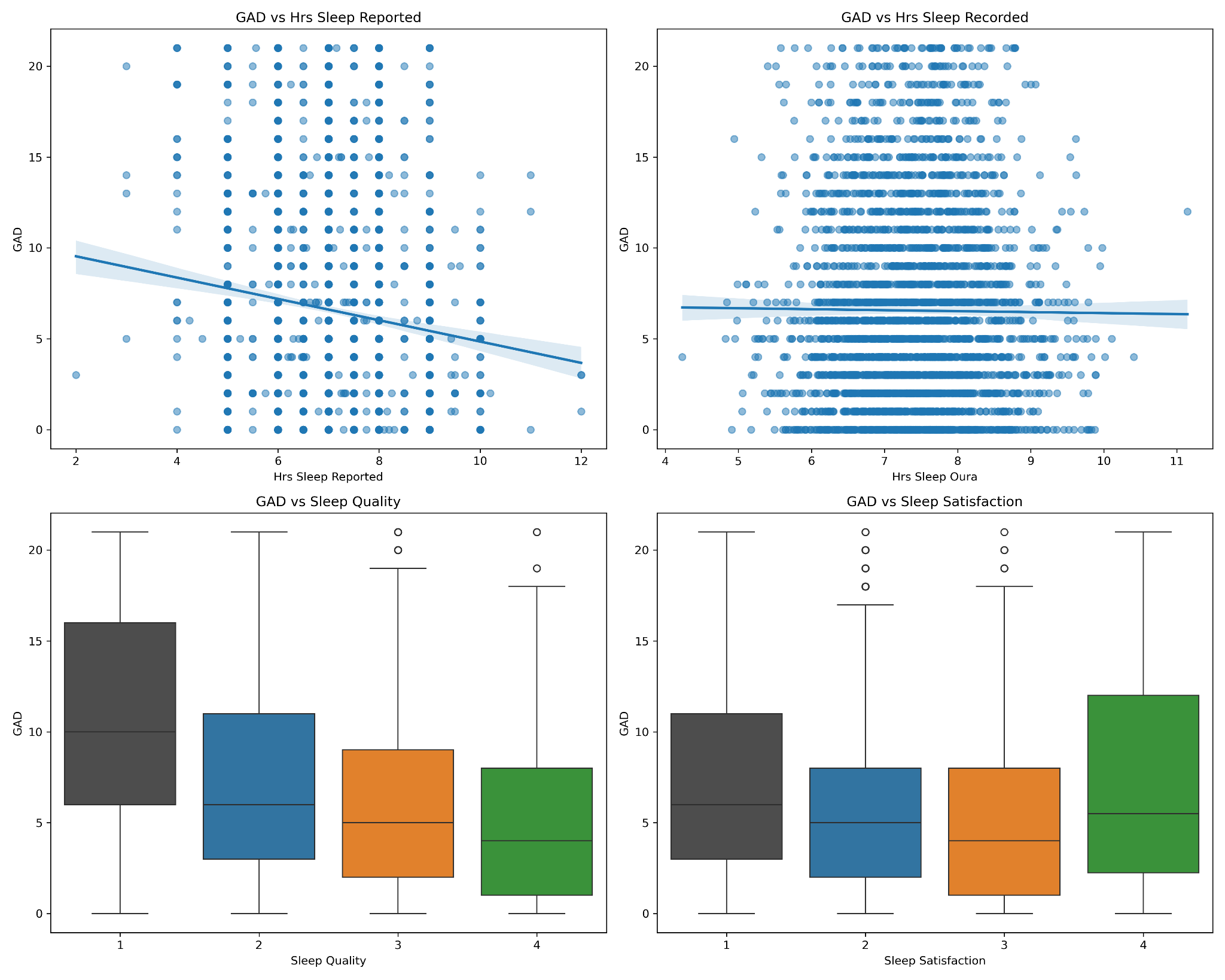
**

**Table S1: Correlation Between Time-Invariant Demographic Predictors and Anxiety**

**Note:** GAD-7 = Generalized Anxiety Disorder Questionnaire; LEC = Life Events Checklist.

a.Spearman rho = 0.232

| Variables | (1) | (2) | (3) | (4) | (5) | (6) | (7) | (8) |
| --- | --- | --- | --- | --- | --- | --- | --- | --- |
| (1) GAD-7 | 1.000 |  |  |  |  |  |  |  |
| (2) Week | -0.061 | 1.000 |  |  |  |  |  |  |
| (3) Non-male gender | 0.255 | 0.028 | 1.000 |  |  |  |  |  |
| (4) Hispanic ethnicity | -0.008 | 0.006 | 0.033 | 1.000 |  |  |  |  |
| (5) First generation | 0.021 | 0.010 | 0.102 | -0.181 | 1.000 |  |  |  |
| (6) Nonwhite | -0.016 | 0.001 | 0.026 | -0.165 | 0.210 | 1.000 |  |  |
| (7) LEC | 0.227 | -0.009 | 0.091 | -0.078 | 0.069 | -0.008 | 1.000 |  |
| (8) Anxiety diagnosis | **0.358** | -0.010 | 0.231 | -0.013 | -0.004 | -0.064 | 0.232 | 1.000 |
|  | | | | | | | | |

**Table S2: Correlation Between Time-Invariant Personality Trait Predictors and Anxiety**

**Note:** GAD-7 = Generalized Anxiety Disorder Questionnaire

a.Spearman rho = 0.150

| Variables | (1) | (2) | (3) | (4) | (5) | (6) |
| --- | --- | --- | --- | --- | --- | --- |
| (1) GAD-7 | 1.000 |  |  |  |  |  |
| (2) Openness | 0.029 | 1.000 |  |  |  |  |
| (3) Conscientiousness | 0.019 | 0.091 | 1.000 |  |  |  |
| (4) Extraversion | 0.102 | **0.309** | 0.035 | 1.000 |  |  |
| (5) Agreeableness | -0.004 | 0.205 | 0.079 | 0.049 | 1.000 |  |
| (6) Neuroticism | **0.442** | 0.166 | 0.168 | 0.127 | 0.150 | 1.000 |
|  | | | | | | |

**Table S3: Correlation Between Time-Varying Predictors and Anxiety**

**Note:** GAD-7 = Generalized Anxiety Disorder Questionnaire

a.Spearman rho = -0.081

| Variables | (1) | (2) | (3) | (4) | (5) | (6) | (7) |
| --- | --- | --- | --- | --- | --- | --- | --- |
| (1) GAD-7 | 1.000 |  |  |  |  |  |  |
| (2) Sleep quantity (hrs.) - Survey | -0.055 | 1.000 |  |  |  |  |  |
| (3) Sleep quantity (hrs.) - Oura Ring | 0.017 | **0.571** | 1.000 |  |  |  |  |
| (4) Poor sleep quality | 0.115 | -0.317 | -0.277 | 1.000 |  |  |  |
| (5) Poor sleep satisfaction | 0.128 | **-0.438** | **-0.311** | **0.423** | 1.000 |  |  |
| (6) Stressful event | **0.403** | -0.096 | -0.071 | 0.127 | 0.125 | 1.000 |  |
| (7) Academic paper or test | 0.030 | -0.064 | -0.020 | 0.124 | 0.061 | -0.081 | 1.000 |
|  | | | | | | | |

**Table S4: Mixed-effects Linear Regression Model**

**Note:** Model form: *GAD_ij_* = (*β*_00_ + *β_01, 02,... 0S_* Time Invariant Predictors*_i_* +*u*_0i_) + (*β*_10_ week_ij_ +*u*_1i_) +  (*β*_20,30…_ s Time VaryingPredictors*_ij_* )+ *ε_ij_*

| **Term** | **Definition** | **Description** |
| --- | --- | --- |
| β_00_ | Intercept fixed effect | Mean score on GAD all individuals when all other values are 0. |
| *β*_01_, _02_,..._0s_ | Coefficient fixed effects for Time-Invariant Predictors | Gender, Race, Ethnicity, First generation status, Personality traits, Mental Health History, Life Events Checklist. |
| u_0i_ | Random effect for intercept | Variation around the intercept across individuals. All participants have a unique intercept. |
| β_1o_ | Fixed effect for week (change) | Association between week and GAD-7 scores. |
| week_ij_ | Predictor for time | Varies across time points and individuals. |
| u_1i_ | Random effect for time | Measures the extent that slope coefficient for time varies across the sample. All participants have their own slope. |
| *β*_20,30… s_ | Coefficient fixed effects for time-varying predictors | Separate slopes that correspond to the intercept for that slope. Reported sleep duration, recorded sleep duration, poor sleep quality, low sleep satisfaction, weekly stressor, academic stressor. |
| ε_ij_ | Residual variance | Varies across time points and individuals. |

**Table S5: Anxiety Scores and Imputed Anxiety Scores for Full Sample (N=556)**

**Note:**  GAD-7 = Generalized Anxiety Disorder Questionnaire; GAD-meanimp = Missing values are replaced by the mean value within each participant; GAD-medianimp = Missing values are replaced by median of the observed values within each participant; GAD-locfimp = Missing values are replaced by last observed value for that variable for each participant; GAD-knnimp = Missing values are replaced by values of nearest neighbors for that variable for each participant.

| Variable | Obs | Mean | Std. Dev. | Min | Max |
| --- | --- | --- | --- | --- | --- |
| GAD-7 | 3159 | 6.495 | 5.332 | 0 | 21 |
| GAD-7 mean imp | 3892 | 6.650 | 5.233 | 0 | 21 |
| GAD-7 median imp | 3892 | 6.626 | 5.292 | 0 | 21 |
| GAD-7 knn imp | 3892 | 6.496 | 5.134 | 0 | 21 |
| GAD-7 linint | 3892 | 6.712 | 5.362 | 0 | 21 |
| GAD-7 locfimp | 3892 | 6.690 | 5.394 | 0 | 21 |
| GAD-7 multimp | 3892 | 6.479 | 4.580 | 0 | 21 |
| GAD-7 predictivemm | 3892 | 6.472 | 4.582 | 0 | 21 |
| GAD-7 survey count | 556 | 5.682 | 1.570 | 1 | 7 |
| GAD-7 study min | 556 | 3.696 | 4.109 | 0 | 21 |
| GAD-7 study max | 556 | 10.077 | 5.649 | 0 | 21 |
| GAD-7 study avg | 556 | 6.650 | 4.664 | 0 | 21 |
| GAD-7 study var | 556 | 6.381 | 4.160 | 0 | 21 |
|  | | | | | |

**Table S6: Anxiety Scores and Imputed Anxiety Scores for Reduced Sample (N=525)**

**Note:**  GAD-7 = Generalized Anxiety Disorder Questionnaire; GAD-meanimp = Missing values are replaced by the mean value within each participant; GAD-medianimp = Missing values are replaced by median of the observed values within each participant; GAD-locfimp = Missing values are replaced by last observed value for that variable for each participant; GAD-knnimp = Missing values are replaced by values of nearest neighbors for that variable for each participant.

| Variable | Obs | Mean | Std. Dev. | Min | Max |
| --- | --- | --- | --- | --- | --- |
| GAD-7 | 3112 | 6.482 | 5.326 | 0 | 21 |
| GAD-7 meanimp | 3112 | 6.456 | 5.274 | 0 | 21 |
| GAD-7 medianimp | 3112 | 6.449 | 5.309 | 0 | 21 |
| GAD-7 knnimp | 3112 | 6.449 | 5.249 | 0 | 21 |
| GAD-7 linint | 3112 | 6.479 | 5.37 | 0 | 21 |
| GAD-7 locfimp | 3112 | 6.484 | 5.382 | 0 | 21 |
| GAD-7 multimp | 3112 | 6.443 | 5.081 | 0 | 21 |
| GAD-7 predictivemm | 3112 | 6.435 | 5.082 | 0 | 21 |
| GAD-7 survey count | 525 | 6.182 | 1.070 | 3 | 7 |
| GAD-7 study min | 525 | 3.442 | 3.816 | 0 | 21 |
| GAD-7 study max | 525 | 9.989 | 5.685 | 0 | 21 |
| GAD-7 study avg | 525 | 6.482 | 4.565 | 0 | 21 |
| GAD-7 study var | 525 | 6.547 | 4.020 | 0 | 21 |
|  | | | | | |

**Table S7: Anxiety Scores and Imputed Anxiety Scores for Restrictive Sample (N=233)**

**Note:**  GAD-7 = Generalized Anxiety Disorder Questionnaire; GAD-meanimp = Missing values are replaced by the mean value within each participant; GAD-medianimp = Missing values are replaced by median of the observed values within each participant; GAD-locfimp = Missing values are replaced by last observed value for that variable for each participant; GAD-knnimp = Missing values are replaced by values of nearest neighbors for that variable for each participant.

| Variable | Obs | Mean | Std. Dev. | Min | Max |
| --- | --- | --- | --- | --- | --- |
| GAD | 1631 | 6.508 | 5.239 | 0 | 21 |
| GAD-7 meanimp | 1631 | 6.51 | 5.218 | 0 | 21 |
| GAD-7 medianimp | 1631 | 6.51 | 5.225 | 0 | 21 |
| GAD-7 knnimp | 1631 | 6.499 | 5.224 | 0 | 21 |
| GAD-7 linint | 1631 | 6.532 | 5.266 | 0 | 21 |
| GAD-7 locfimp | 1631 | 6.532 | 5.269 | 0 | 21 |
| GAD-7 multimp | 1631 | 6.493 | 5.172 | 0 | 21 |
| GAD-7 predictivemm | 1631 | 6.49 | 5.172 | 0 | 21 |
| GAD-7 survey count | 233 | 7 | 0 | 7 | 7 |
| GAD-7 study min | 233 | 3.262 | 3.470 | 0 | 18 |
| GAD-7 study max | 233 | 10.386 | 5.543 | 0 | 21 |
| GAD-7 study avg | 233 | 6.508 | 4.467 | 0 | 20 |

**Table S8: Baseline Demographic Data, Weekly Surveys, and Anxiety Measures for Reduced Samples of Participants**

**Note:** Enrolled participants with complete demographic information that completed three or more surveys (N=525) and those that completed all surveys (N=233).

|  | **Completed 3/7 surveys** | **Completed 7/7 surveys** |
| --- | --- | --- |
| **Baseline demographics (categorical)** | % (N) | % (N) |
| **Gender**  Male  Female  Gender minority | 27.43 (144)  66.29 (348)  6.29 (33) | 24.03 (56)  68.24 (159)  7.73 (18) |
| **Race**  Non-white  American Indian  Asian-Pacific  Biracial/Multi-race  Black  White | 12.38 (65)  0.38 (2)  5.52 (29)  5.71 (30)  0.76 (4)  87.62 (460) | 14.59 (34)  0.00 (0)  6.44 (15)  6.44 (15)  1.72 (4)  85.41 (199) |
| **Ethnicity**  Hispanic  Non-Hispanic | 5.33 (28)  94.67 (497) | 4.72 (11)  95.28 (222) |
| **First generation**  First in family to college  Not first in family to college | 8.95 (47)  91.05 (478) | 9.87 (23)  90.13 (210) |
| ***Life Events Checklist***  LEC = 0  LEC = 1  LEC ≥ 2 | 29.71 (156)  31.81 (167)  38.48 (202) | 33.48 (78)  32.19 (75)  34.33 (80) |
| ***Mental health history***  Mental health diagnosis (Any)  Anxiety Disorder  Depression  ADHD  Anorexia or Bulimia  OCD  PTSD  Panic  Emotional disorder  Bipolar  Alcoholism  Delusions  Psychosis | 43.43 (228)  39.05 (205)  30.10 (158)  10.48 (55)  6.29 (33)  6.29 (33)  3.62 (19)  3.62 (19)  1.14 (6)  0.95 (5)  0.38 (2)  0.00 (0)  0.00 (0) | 41.63 (97)  39.01 (91)  27.90 (65)  6.44 (15)  6.87 (16)  7.30 (17)  3.43 (8)  3.00 (7)  0.86 (2)  0.86 (2)  0.43 (1)  0.00 (0)  0.00 (0) |
| **Baseline traits** | Mean (SD | Mean (SD) |
| ***Personality Trait***  Openness  Conscientiousness  Extraversion  Agreeableness  Neuroticism | 2.52 (0.91)  2.25 (1.06)  3.73 (1.45)  3.02 (1.10)  3.71 (1.25) | 2.69 (0.90)  2.15 (1.02)  3.97 (1.45)  3.00 (1.08)  3.76 (1.22) |
| **Weekly behaviors and events** | Mean (SD | Mean (SD |
| ***Sleep time***  Reported nightly sleep duration (hrs.) - Survey  Recorded nightly total sleep time (hrs.) - Oura Ring | 7.15 (1.08)  7.41 (0.88) | 7.21 (1.02)  7.44 (0.89) |
|  | % Responses | % Responses |
| ***Sleep quality***  Fairly or very good  Poor Sleep  ***Sleep satisfaction***  Too little sleep  Adequate or ideal sleep | 67.18  32.82  45.77  54.23 | 67.23  32.77  46.96  53.04 |
| ***Stressful event***  Yes  No  ***Paper or exam***  Yes  No | 37.72  62.28  24.74  75.26 | 37.16  62.84  24.54  75.46 |
| **Outcome measures** | Mean (SD | Mean (SD) |
| ***Sample level***  GAD-7  ***Individual level***  GAD-7 | 6.48 (5.33)  6.48 (4.57) | 6.51 (5.24)  6.51 (4.47) |
